# Mitochondrial Band-7 family proteins: scaffolds for respiratory chain assembly?

**DOI:** 10.3389/fpls.2014.00141

**Published:** 2014-04-08

**Authors:** Bernadette Gehl, Lee J. Sweetlove

**Affiliations:** Department of Plant Sciences, University of OxfordOxford, UK

**Keywords:** plant mitochondria, respiratory chain, protein complexes, band-7 protein family, cardiolipin, microdomains

## Abstract

The band-7 protein family comprises a diverse set of membrane-bound proteins characterized by the presence of a conserved domain. The exact function of this band-7 domain remains elusive, but examples from animal and bacterial stomatin-type proteins demonstrate binding to lipids and the ability to assemble into membrane-bound oligomers that form putative scaffolds. Some members, such as prohibitins (PHB) and human stomatin-like protein 2 (HsSLP2), localize to the mitochondrial inner membrane where they function in cristae formation and hyperfusion. In *Arabidopsis*, the band-7 protein family has diversified and includes plant-specific members. Mitochondrial-localized members include prohibitins (AtPHBs) and two stomatin-like proteins (AtSLP1 and -2). Studies into PHB function in plants have demonstrated an involvement in root meristem proliferation and putative scaffold formation for mAAA proteases, but it remains unknown how these roles are achieved at the molecular level. In this minireview we summarize the current status of band-7 protein functions in *Arabidopsis*, and speculate how the mitochondrial members might recruit specific lipids to form microdomains that could shape the organization and functioning of the respiratory chain.

## INTRODUCTION

Biological membranes are highly organized proteolipid domains and there is increasing evidence of fine-scale organization into microdomains (Whitelegge, 2011; Holthuis and Ungermann, 2013). These comprise localized assemblies of specific proteins and lipids and are important in the spatial and temporal control of membrane protein complex assembly and regulation. Membrane microdomains are formed by specific protein–protein and protein–lipid interactions that take place within and in the vicinity of membranes, often guided by specialized proteins acting as scaffolds. Some of the most important membrane-integrated protein complexes occur in the inner mitochondrial membrane, which houses the respiratory chain. Despite our increasing knowledge about the composition of respiratory complexes, we have a much less detailed understanding about the dynamics, regulation, and assembly of these complexes at the molecular level within the membrane environment. It is also unclear to what extent scaffold proteins and interactions with specific lipids are involved. In this review we highlight the functions of the band-7 family of scaffold proteins in plants and speculate how a conserved mechanism of self assembly into oligomeric ring structures together with lipid interactions could contribute to the creation of microenvironments within the mitochondrial inner membrane.

## BAND-7 FAMILY PROTEINS: MOLECULAR SCAFFOLD PROTEINS WITH DIVERSE FUNCTIONS

The band-7 protein family comprises a diverse set of prokaryotic and eukaryotic membrane proteins characterized by the presence of a conserved “band-7” domain in the central regions of the protein sequence. The domain name stems from the first identified member, human stomatin (or erythrocyte band 7.2b protein). The superfamily is also known as “SPFH” according to the initials of its members (stomatin, prohibitin,flotillin, HflC/K; Tavernarakis et al., 1999). Band-7 family proteins generally form oligomers and regulate the assembly and activity of super-molecular protein complexes in various cellular localizations, often linked to membrane microdomains (Browman et al., 2007). Despite its high degree of conservation, the precise function of the band-7 domain remains unknown in most organisms. Over the years numerous examples have emerged demonstrating regulation of various ion channels and transporters by stomatins (Price et al., 2004; Huber et al., 2006; Montel-Hagen et al., 2008). Prohibitins (PHB) function in mitochondrial cristae formation (Merkwirth et al., 2008), flotillins are lipid raft markers involved in trafficking events in animal cells (Glebov et al., 2006), and prokaryotic HflC/K type proteins regulate the activity of membrane-bound proteases (Kihara et al., 1996).

## YEAST AND ANIMAL MITOCHONDRIAL BAND-7 PROTEINS ARE INVOLVED IN THE TURNOVER OF MEMBRANE PROTEINS AND AFFECT RESPIRATORY CHAIN ORGANIZATION AND MITOCHONDRIAL MORPHOLOGY

A small subset of eukaryotic band-7 proteins is localized to mitochondria. These include PHBs and mammalian stomatin-like protein 2 (SLP2), both of which have been implicated in regulating the activities of mitochondrial metalloproteases, thereby affecting processes such as cristae formation and respiratory chain assembly (Steglich et al., 1999; Da Cruz et al., 2008; Merkwirth et al., 2008; Tondera et al., 2009).

The first native band-7 protein complex studied was the yeast PHB complex. This large complex (1.2 MDa) is composed of PHB1 and PHB2 units arranged as an oligomeric ring of 16–20 nm diameter and is associated with the mitochondrial inner membrane facing the intermembrane space (Nijtmans et al., 2000; Tatsuta et al., 2005). A second PHB complex was discovered in yeast that additionally contains a matrix-exposed AAA protease (Steglich et al., 1999). AAA-type proteases belong to the metalloprotease family and contain an additional ATP-hydrolysing domain. They are thought to function in membrane protein quality control (Langer, 2000). PHB in yeast was found to negatively regulate matrix-AAA (mAAA) activity, thereby influencing turnover rates of mitochondrial-encoded respiratory chain subunits (Steglich et al., 1999). Transient associations of PHBs with cytochrome c oxidase subunits were demonstrated in yeast, implying that the PHB complex has chaperone functions in complex IV assembly (Nijtmans et al., 2000). Mammalian PHBs were also shown to interact with complex IV subunits, as well as with subunits of complex I (NADH dehydrogenase; Bourges et al., 2004; Schleicher et al., 2008; Strub et al., 2010). Knockdown of PHBs in mouse cells also affects mitochondrial morphology because of altered proteolytic processing of the inner membrane GTPase OPA1 (optical atrophy 1) by metalloproteases (Merkwirth et al., 2008).

Detailed insight into band-7 protein complex formation was gained from a cystallography study of the conserved stomatin domain from mouse (Brand et al., 2012). The basic unit in the crystal was found to be a banana-shaped dimer capable of forming a ring-shaped structure required for stomatin function in ion channel modulation. A ring structure was also observed by single particle analysis of a purified stomatin complex from cyanobacteria (Boehm et al., 2009), making it likely that other band-7 family proteins might also adapt this shape as assembled complexes. A related stomatin-like protein from human and rodents, SLP2 (stomatin-like protein 2), also forms a large (1.8 MDa) complex in mitochondria (Reifschneider et al., 2006). Notably, no ortholog of mammalian SLP2 is present in the yeast *Saccharomyces cerevisiae*. Mammalian SLP2 is peripherally associated with the mitochondrial inner membrane on the side of the intermembrane space (Hajek et al., 2007; Da Cruz et al., 2008), where it forms a complex with mitofusin-2 (Mfn-2), a GTPase of the outer membrane mediating mitochondrial fusion. Mammalian SLP2 also interacts with PHBs in a smaller 250 kDa complex (Da Cruz et al., 2008). Knockdown of SLP2 in HeLa cells caused increased proteolysis of PHBs and respiratory chain subunits from complexes I and IV by metalloproteases (Da Cruz et al., 2008), as well as a reduction in membrane potential, but had no effect on mitochondrial morphology (Hajek et al., 2007). By contrast, SLP2 knockdown in mice was reported to be embryo lethal (Christie et al., 2012), the same as knockouts of PHBs in mice (Merkwirth et al., 2008). A T-cell specific knockdown of SLP2 caused a reduction of complex I (NADH dehydrogenase) subunits and reduced complex I activity (Christie et al., 2012). Interestingly, human recombinant SLP2 was demonstrated to bind preferentially to cardiolipin (CL) in an *in vitro* pull-down assay that utilized liposomes with varying phospholipid composition (Christie et al., 2011), although the specificity of this interaction is debatable because the assay lacked additional control proteins. Additionally, yeast genetic studies have revealed that enzymes involved in CL and phosphatidylethanolamine (PE) synthesis pathways are essential for survival in *phb* knockout strains, underlining a functional link between lipid synthesis and PHBs (Birner et al., 2003; Osman et al., 2009).

## PLANT BAND-7 PROTEINS

The band-7 protein family is more diverse in higher plants than it is in yeast and animals, and includes a plant-specific group of proteins classified as HIR (hypersensitive response induced), as well as a larger number of PHBs and two stomatin-like proteins (Nadimpalli et al., 2000). This large diversity in higher plants is down to gene duplications and may be linked to the requirement to adapt to environmental stress conditions (Van Aken et al., 2010).

**FIGURE 1:**
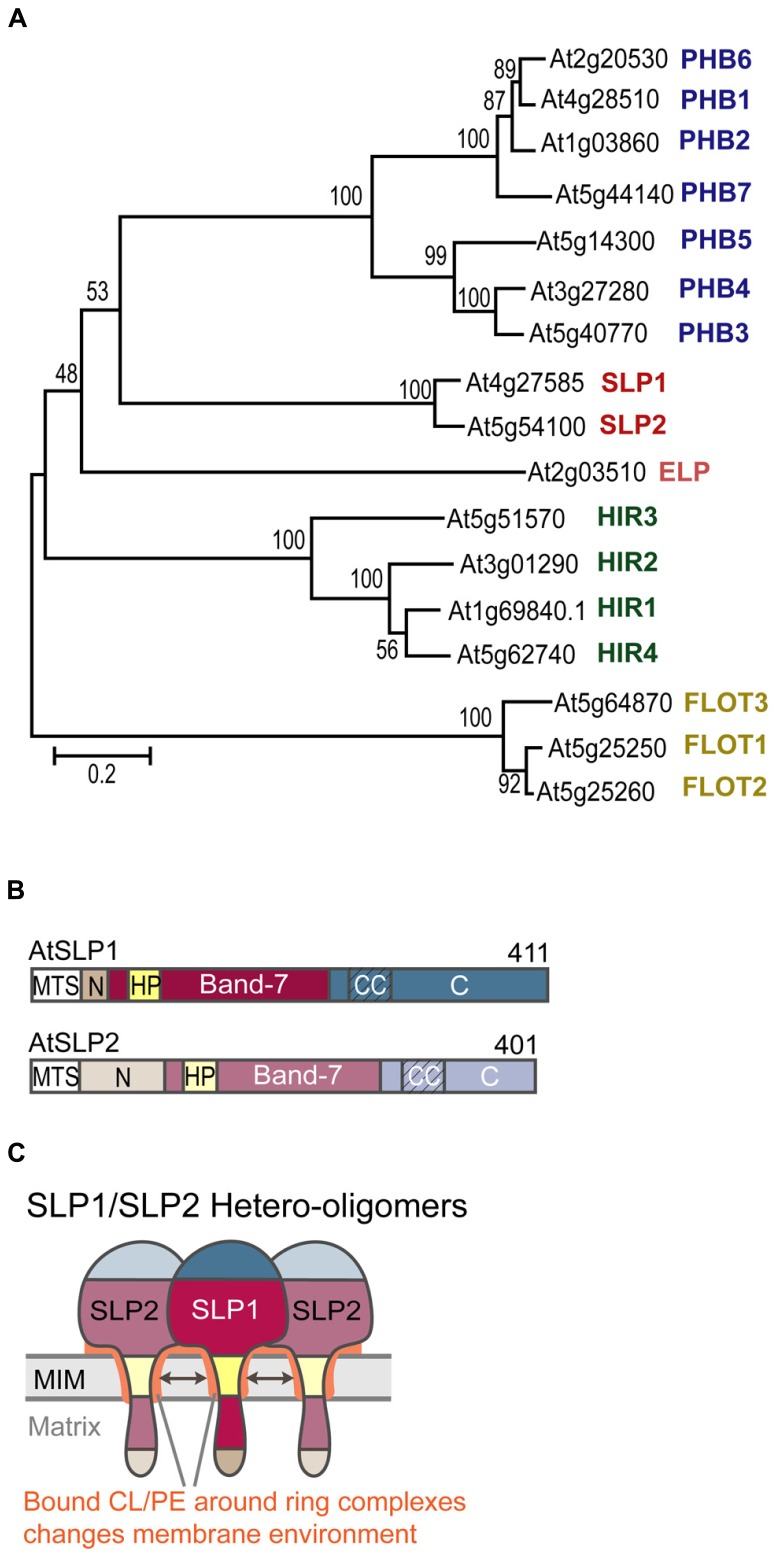
***Arabidopsis thaliana* band-7 protein family members. (A)** Phylogenetic bootstrap tree of the *Arabidopsis* band-7 protein family. PHBs (prohibitins) in blue, SLPs (stomatin-like proteins) in red, ELP (erlin-like protein) in pink, HIRs (hypersensitive response induced proteins) in green, FLOTs (flotillin-like proteins) in yellow. This tree was created by the neighborhood-joining method. Reprinted with permission by ASPB. **(B)** Schematic of AtSLP domains. MTS (mitochondrial targeting sequence), N (N-terminal domain), HP (hydrophobic patch), Band-7 (band-7 domain), CC (predicted coiled-coil regions), C (C-terminal domain). **(C)** Schematic of SLP1-SLP2 hetero-oligomers embedded within the mitochondrial inner membrane. Coloring corresponds to the scheme in **B**. Cardiolipin-binding to SLP oligomers is indicated by the orange color around the hydrophobic patches and between the inner membrane and the band-7 domains.

The *Arabidopsis* genome encodes 17 genes that contain the band-7 domain (InterPro IPR001107). The gene products fall into five distinct classes based on sequence homologies with animal and yeast orthologs: seven genes belong to the PHBs, of which five are expressed, two are stomatin-like (AtSLP1 and -2), four belong to the HIR proteins (HIRs1-4), (Qi et al., 2011), three resemble flotillins, and one protein has similarities to erlin proteins from animals (Browman et al., 2007; **Figure 1A**). *Arabidopsis* band-7 proteins are found in various subcellular membrane localizations according to the SUBA database (Heazlewood et al., 2007). Most of these locations are based on mass spectrometry data from various proteomics studies, but for PHBs, SLPs and HIRs, additional *in vivo* data from fluorescent tagging experiments are available (Marmagne et al., 2004; Van Aken et al., 2007, 2009; Qi et al., 2011; Gehl et al., 2014). According to these data, *Arabidopsis* PHBs are primarily localized to mitochondria (Van Aken et al., 2007), although localization to the cytoplasm and the nucleus was suggested in a separate study (Christians and Larsen, 2007). However, this result is controversial because cytoplasmic localization was not confirmed by any additional cytosolic markers. AtSLPs are found exclusively in mitochondria (Gehl et al., 2014), whereas HIRs are localized to the plasma membrane (Qi et al., 2011). Although nothing is known about the functions of the three flotillin-like proteins or the erlin-like protein, forward and reverse genetics studies have started to elucidate the molecular roles of HIRs, PHBs, and SLPs.

## PLANT MITOCHONDRIAL BAND-7 MEMBERS

In plants, much less is known about the specific functions of SLPs and PHBs in mitochondria and how the complexes they form relate to each other. Research into plant PHBs and SLPs so far suggests that the two complexes are physically distinct and are not functionally redundant, yet they seem to share several functions with animal and yeast orthologs.

The best-studied plant band-7 family proteins are the PHBs. The seven *Arabidopsis* PHBs fall into two classes (type I or II) according to similarities with yeast and animal PHB1 and PHB2 (Van Aken et al., 2007, 2010). Reverse genetics has revealed diverse functions for plant PHBs (Van Aken et al., 2007). Knockout of *AtPHB3* resulted in retarded growth of roots and shoots which was linked to reduced cell division and expansion in apical meristems, as well as alterations in mitochondrial morphology, indicative of a lack of cristae. By contrast, knockout of *AtPHB4* did not result in any obvious growth phenotypes, but a double *phb3/4* mutant was lethal. From this study it was concluded that AtPHBs are important to sustain increased metabolic demands related to cell division in meristems supporting differentiation in apical tissues.

A mutant in the *phb3* gene was also identified as *eer3-1*, a loss-of function conditional point mutation allele leading to an extreme constitutive ethylene response in etiolated seedlings (Christians and Larsen, 2007). An independent loss-of function allele, *phb3-3* was identified in a mutagenesis screen for deficiencies in hydrogen peroxide-induced nitric oxide (NO) accumulation (Wang et al., 2010). This point mutation was mapped to a glycine to aspartate change inside the conserved band-7 domain, but it remains unknown what effect this mutation has on PHB complex formation. Both *phb3-3* and an independent T-DNA knockout allele showed NO-related phenotypes and increased resistance to high salinity, pointing toward yet unknown functions of PHB3 in NO homeostasis, possibly via the respiratory chain.

Tandem-affinity purification of tagged PHB3 revealed that it interacts with all other expressed PHBs (1, 2, 3, 4, and 6), as well as some enzymes and proteins of unknown function (Van Aken et al., 2007). Some class II PHBs have also been found associated with subcomplexes of complex I, possibly as contaminants in mass spectrometry studies (Klodmann et al., 2010). The native PHB complex was later characterized by two-dimensional blue native and SDS-PAGE (Piechota et al., 2010). *Arabidopsis* PHBs form a hetero-oligomeric complex of 1 MDa, but they also participate in a 2 MDa complex together with the mAAA proteases FtsH3 and FtsH10. No complex containing a FtsH protease without PHBs could be identified, suggesting that the PHB complex acts as a scaffold to stabilize the FtsH oligomeric complexes. Other studies of PHBs in Petunia flowers and in tobacco leaves indicated links to cellular senescence, reactive oxygen species production and mitochondrial morphology (Chen et al., 2005; Ahn et al., 2006). Based on these findings, PHBs were suggested to act as universal scaffolds in the mitochondrial inner membrane, likely associated with lipid microdomains that affect a variety of mitochondrial processes (Van Aken et al., 2010).

The two other mitochondrial band-7 family members, stomatin-like proteins (SLPs) were established as mitochondrial proteins in various proteomics studies (Millar et al., 2001; Heazlewood et al., 2004; Dunkley et al., 2006). SLP1 has also been identified in detergent-resistant membrane fractions thought to be derived from the plasma membrane (Borner et al., 2005). The AtSLP1 protein was also shown to be capable of binding to Zn^2^^+^ (Tan et al., 2010) and is threonine-phosphorylated within its hydrophilic C-terminus (Ito et al., 2009). Both AtSLP transcripts were upregulated in the *phb3* mutant, and AtSLP2 has been identified as a stress-responsive gene in a number of microarray experiments (Van Aken et al., 2009).

Our own work has dealt with functionally characterizing AtSLP1 (At4g27585) and AtSLP2 (At5g54100). Both AtSLPs have one conserved band-7 domain, as well as one hydrophobic stretch located within this conserved domain (**Figure 1B**). We have identified a class II (-3R) mitochondrial targeting sequence (MTC) in both SLP sequences, which, upon cleavage, results in a short N-terminal sequence that is probably located in the mitochondrial matrix. The bulk of the proteins likely reside in the intermembrane space. Mature SLP1 is slightly longer (368 amino acids) than SLP2 (360 amino acids), and it possesses a unique hydrophilic C-terminus not present in SLP2 that harbors the phosphorylation site (Ito et al., 2009).

We have localized SLP1 to a large protein complex (3 MDa) in the mitochondrial inner membrane where it most likely interacts with AtSLP2, possibly organized in a ring shape. Sequence homologies suggest that AtSLPs are the plant orthologs of animal SLP2. Knockout of AtSLP1, but not AtSLP2 affects the abundance of complex I and related supercomplexes, but not other respiratory complexes (Gehl et al., 2014). We interpret this specific effect on complex I either as a consequence of deficient complex I assembly, or an increased complex I turnover that is mediated by proteases (likely of the AAA-type) in the inner membrane.

We also hypothesize that complex I deficiency in the absence of SLP1 is related to changes that occur in the local membrane environment. The sequence homology of AtSLPs with human SLP2 may suggest that *Arabidopsis* SLPs can also bind to specific mitochondrial inner membrane lipids such as CL and PE. Lipid-binding could occur at residues located within the hydrophobic SLP membrane anchor, and may help stabilize the membrane anchorage of the SLP oligomers. Additionally, residues found within the band-7 domain could bind to lipids, possibly to keep the SLP complex in close proximity to the inner membrane (**Figure 1C**). This scenario resembles binding of cholesterol by the stomatin proteins podocin and *C. elegans* Mec-2 to the N-terminal hydrophobic domains and the band-7 domain (Huber et al., 2006). Cholesterol binding was mapped to, a conserved proline residue located just upstream of the band-7 domain that proved to be crucial for ion channel regulation by Mec-2.

In a similar manner, the plant PHB complex is likely to bind lipids (CL and/or PE) and assembles into a ring-shaped structure, with the C-termini facing the intermembrane space. Protein interaction data derived from PHBs so far suggest no physical associations between SLPs and PHBs in *Arabidopsis* (Van Aken et al., 2007; Piechota et al., 2010), and it is not clear how the two complexes relate to each other functionally. Because of their different gene expression patterns in *Arabidopsis* based on microarray data and on promoter-GUS fusion plants (Van Aken et al., 2007; Gehl et al., 2014) and the differing growth phenotypes of knockout mutants, we conclude that PHBs and SLPs are not functionally redundant. For example, class I PHBs are highly expressed in root meristem tissue and single* phb3* knockout mutants have a dwarfed growth phenotype with short roots, whereas class I double *phb* mutants are embryo lethal. By contrast, *SLP* genes are not highly expressed in root meristems, and *slp1/2* double knockout plants are viable and do not show abnormal growth morphology (Gehl et al., 2014). Currently nothing is known about respiratory chain function and the abundance of supercomplexes in *phb* mutants.

## DO PLANT PROHIBITINS AND STOMATIN-LIKE PROTEINS COOPERATE IN RESPIRATORY CHAIN ASSEMBLY?

**FIGURE 2:**
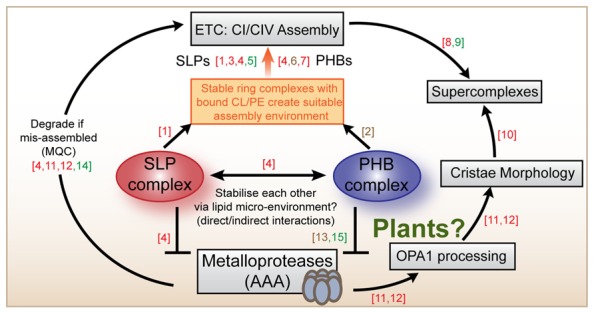
**Summary scheme of SLP and PHB functions across species.** The SLP and the PHB complexes are localized to the mitochondrial inner membrane where they probably bind to cardiolipin and/or phosphatidylethanolamine and participate in the assembly of complexes I and complex IV. In animal cells, SLPs and PHBs have been located to the same complexes (250 kDa) and have been demonstrated to interact. Both are implied to inhibit the activities of chaperone-like proteases of the AAA type which are also embedded in the inner membrane. PHBs from plants and from yeast form a complex with mAAA proteases. AAA proteases are known to participate in mitochondrial quality control (MQC) mechanisms that ensure appropriate electron transport chain (ETC) assembly and functioning. Mammalian AAA proteases are also known to participate in the proteolytic processing of OPA-1 that determines cristae morphology in animal cells. Mitochondrial morphology itself was recently demonstrated to influence the assembly status of supercomplexes in mouse cells. Literature references are as follows: (1) Christie et al. (2011), (2) Osman et al. (2009), (3) Christie et al. (2012), (4) Da Cruz et al. (2008), (5) Gehl et al. (2014), (6) Nijtmans et al. (2000), (7) Bourges et al. (2004), (8) Ac’i (2008), (9) Eubel et al. (2003), (10) Cogliati et al. (2013), (11) Merkwirth et al. (2008), (12) Tondera et al. (2009), (13) Steglich et al. (1999), (14) Kolodziejczak et al. (2007), (15) Piechota et al. (2010). Coloring indicates which model system was studied: red (animals), brown (yeast), green (plants).

We suggest that a possible solution to the apparent functional specificity of SLPs and PHBs despite overlapping properties of the proteins could be that the two complexes cooperatively connect mitochondrial quality control (MQC) by proteases with respiratory chain assembly (**Figure 2**). Evidence from our work and from mice and HeLa cells points toward a specific function for the SLP complex in the assembly and/or turnover of complex I (Da Cruz et al., 2008; Christie et al., 2012; Gehl et al., 2014). Studies into yeast and mouse PHBs suggest that the PHB complex likely functions in the assembly or turnover of complex IV (Nijtmans et al., 2000; Strub et al., 2010), although other studies imply that it is also related to complex I (Bourges et al., 2004; Acín-Pérez et al., 2008). Cardiolipin-binding by SLP and PHB ring complexes could directly affect the formation of functional supercomplexes which are known to be dependent on the incorporation of multiple CL molecules internally and at the interphases between complexes (Bazan et al., 2013; Pineau et al., 2013). The SLP and PHB complexes likely change their local membrane environment by specifically sequestering CL and/or PE, possibly by forming a localized network of rings that helps respiratory chain assembly at specific sites. This could take place in a coordinated fashion between both complexes, such that both rings affect the membrane environment and each other by altering the tension, charge distribution and possibly even curvature of the inner membrane. Membrane-bound AAA-proteases likely contribute to turnover rates of respiratory chain components as part of MQC. In animal cells metalloproteases also help processing OPA1, thereby determining cristae ultrastructure and mitochondrial morphology (Merkwirth et al., 2008; Tondera et al., 2009). Recently, cristae morphology governed by OPA1 processing has also been linked to the assembly status of supercomplexes, although a direct connection between OPA1 and supercomplexes is so far missing (Cogliati et al., 2013). In plants, no OPA1-like protein exists and the mechanism determining cristae morphology remains unknown.

In summary, we place SLP and PHB complexes at the heart of a mechanism that incorporates AAA proteases and phospholipids, thereby affecting respiratory chain function at the point of assembly and turnover. We hypothesize that CL/PE binding by SLPs/PHBs creates defined areas of respiratory chain assembly and quality control. Currently this theory is speculative, but could be addressed experimentally. Firstly, a detailed inventory about defects in the respiratory chain in the respective *Arabidopsis* mutant backgrounds is needed, in combination with an analysis of the lipid-binding properties of AtPHBs and AtSLPs. These results, together with complementary structure-function approaches and high resolution imaging techniques will give new insights into the extent of cooperation between these protein complexes and will clarify where their specificities lie. This information will not only advance our understanding of inner membrane compartmentation, but also help to elucidate band-7 protein function throughout the kingdoms.

## Conflict of Interest Statement

The authors declare that the research was conducted in the absence of any commercial or financial relationships that could be construed as a potential conflict of interest.
